# Targeting Acute Myeloid Leukemia Using the RevCAR Platform: A Programmable, Switchable and Combinatorial Strategy

**DOI:** 10.3390/cancers13194785

**Published:** 2021-09-24

**Authors:** Enrico Kittel-Boselli, Karla Elizabeth González Soto, Liliana Rodrigues Loureiro, Anja Hoffmann, Ralf Bergmann, Claudia Arndt, Stefanie Koristka, Nicola Mitwasi, Alexandra Kegler, Tabea Bartsch, Nicole Berndt, Heidi Altmann, Frederick Fasslrinner, Martin Bornhäuser, Michael Philipp Bachmann, Anja Feldmann

**Affiliations:** 1 Department of Radioimmunology, Institute of Radiopharmaceutical Cancer Research, Helmholtz-Zentrum Dresden-Rossendorf (HZDR), 01328 Dresden, Germany; enrico.k-b@maboss.de (E.K.-B.); k.gonzales@hzdr.de (K.E.G.S.); l.loureiro@hzdr.de (L.R.L.); anjhoff@onlinehome.de (A.H.); r.bergmann@hzdr.de (R.B.); c.arndt@hzdr.de (C.A.); stefanie.koristka@gmx.de (S.K.); n.Metwasi@hzdr.de (N.M.); a.kegler@hzdr.de (A.K.); t.bartsch@hzdr.de (T.B.); n.berndt@hzdr.de (N.B.); a.feldmann@hzdr.de (A.F.); 2 Tumor Immunology, University Cancer Center (UCC), University Hospital Carl Gustav Carus Dresden, TU Dresden, 01307 Dresden, Germany; 3 Department of Biophysics and Radiation Biology, Semmelweis University, 1094 Budapest, Hungary; 4 Mildred Scheel Early Career Center, Faculty of Medicine Carl Gustav Carus, TU Dresden, 01307 Dresden, Germany; Frederick.Fasslrinner@uniklinikum-dresden.de; 5 Medical Clinic and Polyclinic I, University Hospital Carl Gustav Carus, TU Dresden, 01307 Dresden, Germany; Heidi.Altmann@uniklinikum-dresden.de (H.A.); Martin.Bornhaeuser@uniklinikum-dresden.de (M.B.); 6 National Center for Tumor Diseases (NCT), 01307 Dresden, Germany; 7 Faculty of Medicine, University Hospital Carl Gustav Carus, TU Dresden, 01307 Dresden, Germany; 8 German Cancer Research Center (DKFZ), 69120 Heidelberg, Germany; 9 German Cancer Consortium (DKTK), Partner Site Dresden, 01307 Dresden, Germany

**Keywords:** chimeric antigen receptor (CAR), tumor immunotherapy, combinatorial gated targeting, acute myeloid leukemia (AML)

## Abstract

**Simple Summary:**

Acute myeloid leukemia (AML) is a type of blood malignancy particularly affecting the myeloid lineage and one of the most common types of leukemia in adults. It is characterized by high heterogeneity among patients leading to immune escape and disease relapse, which challenges the development of immunotherapies such as chimeric antigen receptor (CAR) T-cells. In this way, the aim of our work was to establish the modular RevCAR platform as a combinatorial tumor targeting approach for the treatment of AML. Herein, we demonstrate the preclinical flexibility and efficiency of RevCAR T-cells in targeting patient-derived AML cells expressing CD33 and CD123. Furthermore, AND gate logic targeting these antigens was successfully established using the RevCAR platform. These accomplishments pave the way towards the future clinical translation of such an improved and personalized immunotherapy for AML patients aiming long-lasting anticarcinogenic responses.

**Abstract:**

Clinical translation of novel immunotherapeutic strategies such as chimeric antigen receptor (CAR) T-cells in acute myeloid leukemia (AML) is still at an early stage. Major challenges include immune escape and disease relapse demanding for further improvements in CAR design. To overcome such hurdles, we have invented the switchable, flexible and programmable adaptor Reverse (Rev) CAR platform. This consists of T-cells engineered with RevCARs that are primarily inactive as they express an extracellular short peptide epitope incapable of recognizing surface antigens. RevCAR T-cells can be redirected to tumor antigens and controlled by bispecific antibodies cross-linking RevCAR T- and tumor cells resulting in tumor lysis. Remarkably, the RevCAR platform enables combinatorial tumor targeting following Boolean logic gates. We herein show for the first time the applicability of the RevCAR platform to target myeloid malignancies like AML. Applying in vitro and in vivo models, we have proven that AML cell lines as well as patient-derived AML blasts were efficiently killed by redirected RevCAR T-cells targeting CD33 and CD123 in a flexible manner. Furthermore, by targeting both antigens, a Boolean AND gate logic targeting could be achieved using the RevCAR platform. These accomplishments pave the way towards an improved and personalized immunotherapy for AML patients.

## 1. Introduction

**Figure 1:**
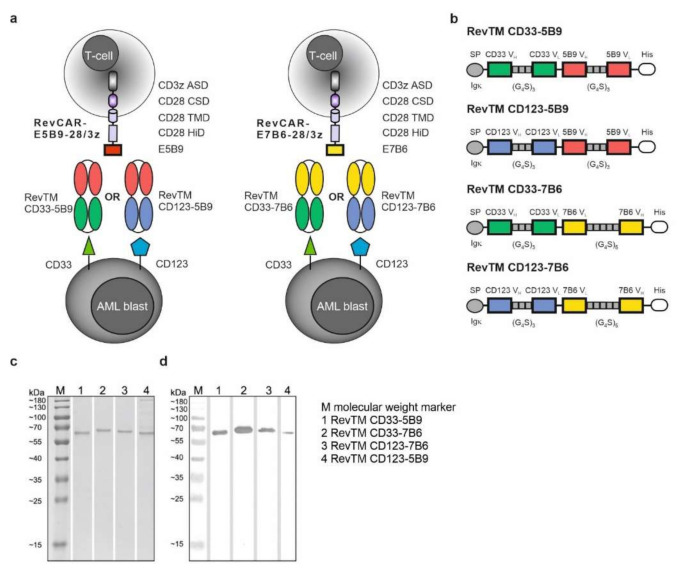
**RevCAR platform targeting CD33 and CD123.** (**a**) Schematic overview of the RevCAR system and (**b**) design of the RevTMs. (**a**) RevCARs consist of the extracellular peptide epitope E5B9 or E7B6 and CD28 (28) hinge domain (HiD), CD28 transmembrane domain (TMD), the intracellular CD28 costimulatory (CSD) and CD3 zeta (3z) activating signaling domain (ASD). RevCAR-E5B9-28/3z or RevCAR-E7B6-28/3z modified T-cells are redirected towards CD33 or CD123 expressed on AML blasts via adaptor target modules, named RevTMs. RevTMs are bispecific antibodies (bsAbs) consisting of two different single-chain fragments variable (scFvs) binding on the one hand to E5B9 or E7B6 of the RevCAR and on the other hand to CD33 or CD123 on the surface of AML blasts. (**b**) In detail, the scFvs of the RevTMs are constructed with the variable heavy (V_H_) and light chain (V_L_) domains derived from the monoclonal antibodies (mAbs) CD33, CD123, 5B9, or 7B6 connected via glycine (G)-serine (S) linkers. RevTMs are expressed in eukaryotic cell lines and secreted into the cell culture supernatant mediated by the Ig kappa leader sequence (Igk). After purification via histidine tag (His), RevTMs were separated by SDS-PAGE and analyzed using Coomassie staining (**c**) and immunodetection after blotting on nitrocellulose membrane via anti-His Ab and AP-conjugated anti-mouse Ab (**d**). The whole western blot figures can be found in Figure S3.

Treatment of acute myeloid leukemia (AML) is still challenged by inadequate long-lasting anticarcinogenic responses due to its heterogeneity and poorly known resistance mechanisms [1]. Due to the lack of beneficial outcomes for the patients that undergo conventional therapy, which mainly comprises chemotherapy usually followed by stem cell transplantation [2], there is an urgent need for innovative strategies. Thus, in the past years, the treatment of AML has been significantly shifted towards the development of targeted approaches [3]. Current targeted strategies clinically implemented mainly include the administration of gemtuzumab ozogamicin (GO), an antibody (Ab)-drug conjugate targeting the surface antigen CD33 [4]. Alternatively, other promising approaches such as novel T-cell-based immunotherapies have shown better outcomes for the treatment of acute lymphoblastic leukemia (ALL), lymphoma and multiple myeloma [5]. Examples of such successful clinical translation are CD19- and BCMA-specific CAR T-cells [6,7,8,9]. However, preclinical and early clinical trials using targeted immunotherapeutic approaches demonstrated the challenging setting of AML, in which relapse driven by residual leukemic stem cells (LSCs), tumor escape variants as well as the lack of suitable targetable surface antigens are the main obstacles. Even though an ideal target has not yet been identified, CD33 and CD123 are promising candidates being expressed on AML bulk cells and LSCs at initial diagnosis and relapse cases. Normally, AML cells express CD33, CD123, or even both, while lack of expression of both antigens (CD33 and CD123) is extremely rare [10,11]. Given this, the enthusiasm to exploit CAR T-cell-based technologies targeting these antigens is reflected by the promising number of early clinical trials currently ongoing. In this regard, an impressive and promising phase I clinical trial using UniCAR T-cells targeting CD123 for the treatment of CD123-positive hematologic and lymphoid malignancies (NCT04230265) proved to be a breakthrough. These promising results have demonstrated functionality and switchability of adaptor CAR T-cell systems for the first time in humans [12]. Given the heterogeneity of antigen expression on AML cells, combinatorial targeting approaches are required as an improved therapeutic tool. Bearing this in mind, and based on our experience working with conventional CAR T-cells [13] and modular Ab-based systems to redirect T-cells [14], we developed the adaptor Reverse CAR (RevCAR) platform [15]. This was particularly developed based on the established UniCAR system [16,17,18,19,20], aiming controllable and programmable combinatorial tumor targeting to improve tumor specificity and safety of CAR T-cell therapy. Particularly, RevCAR T-cells are engineered to express RevCARs that consist of the peptide epitopes E5B9 or E7B6 instead of a single-chain fragment variable (scFv) that is commonly used as extracellular domain in conventional second-generation CARs. Due to the lack of an antigen-binding motif, RevCARs do not recognize a tumor antigen and thus are primarily inactive. Cross-linkage between RevCAR T-cells and tumor cells is exclusively mediated by bispecific target modules (RevTMs). These RevTMs consist of two scFvs, one targeting the respective RevCAR E5B9 or E7B6 epitope and the other one the tumor antigen of interest (Figure 1a). The modular feature of this system allows the steering and rapid on/off switch of the RevCAR T-cell activity by dosing of the RevTMs, avoiding side-effects like on-target/off-tumor toxicity and cytokine release syndrome (CRS) commonly reported for conventional CAR T-cell approaches [21,22]. The small size of the RevCAR epitope molecules, with the E5B9 epitope consisting of 10 amino acids (aa) or the E7B6 of 18 aa, accordingly reduce the length of the RevCAR encoding genes. These features are of considerable value allowing the expression of multiple CAR genes using a single construct. Consequently, we have successfully generated Dual-RevCAR T-cells that can be used for targeting of prostate tumor cells following the Boolean logic gating [15]. These Dual-RevCAR T-cells express two separate RevCARs with different specificities, one RevCAR transmitting the activation signal and the other one mediating the costimulatory signal upon recognition of different antigens on the same tumor cell.

Given the demonstrated potential of the Dual-RevCARs and bearing in mind the challenges faced by immunotherapies targeting AML, we adapted the RevCAR system to target such prevalent myeloid malignancy aiming for an advanced CAR T-cell control, improved targeting specificity, efficient killing of tumor escape variants and reduction of on-target/off-tumor toxicity. We hereby demonstrate efficient activation of RevCAR T-cells and specific killing of AML cells and blasts expressing the markers CD33 and/or CD123 both in vitro and in vivo. In order to overcome tumor escape variants, universal RevCAR T-cells can be flexibly adapted to target CD33 or CD123 AML markers without re-engineering of T-cells. Moreover, we provide proof-of-concept of AND gate logic targeting of AML cells and blasts using the Dual-RevCAR platform.

## 2. Materials and Methods

### 2.1. Cell Lines

HEK-293T and 3T3 cells were obtained from ATCC (Manassas, VA, USA), while MOLM-13 and OCI-AML3 were purchased from DSMZ (Braunschweig, Germany) without further authentication. For in vivo and in vitro studies, MOLM-13 cells were genetically modified to express the firefly Luciferase (Luc) using lentiviral transduction and termed MOLM-13-Luc. OCI-AML3, HEK-293T and 3T3 cells were kept in DMEM complete, while MOLM-13 and MOLM-13-Luc were cultured in RPMI 1640 complete [14,15] supplemented with 20% fetal bovine serum (FBS). All cells were maintained at 37 °C in a humidified atmosphere of 5% CO_2_. AML cell lines were cultured for a time period not longer than three months. All cells were regularly tested for Mycoplasma infection.

### 2.2. Isolation of Human Peripheral Blood Mononuclear Cells (PBMCs), Patient-Derived AML Blasts and Lentiviral Transduction of Human T-Cells

Primary T-cells were obtained from buffy coats (German Red Cross, Dresden, Germany) of healthy voluntary donors provided by the German Red Cross (Dresden, Germany) after written consent of the donors. The research with human T-cells was approved by the local ethics committee of the Medical Faculty Carl Gustav Carus, Technical University Dresden, Germany (EK27022006). Isolation and cultivation of PBMCs and T-cell subpopulations were performed as previously described [14,15,18]. In a similar way, patient-derived mononuclear cells (MNCs) containing high percentage of AML blasts were isolated from bone marrow aspirates or peripheral blood from newly diagnosed patients and termed patient-derived AML blasts. These were cultured in StemSpanTM SFEM media (StemCell, Cologne, Germany) supplemented with FBS, penicillin, streptomycin, glutamine, fms-related tyrosine kinase 3 ligand (FLT3-L), stem cell factor, thrombopoietin (TPO) and interleukin-3 (IL-3). The respective AML patients were recruited within the Study Alliance Leukemia (SAL) registry. Written informed consent has been obtained and the institutional ethics review board has approved the SAL AML registry and integrated biobank (EK98032010). After isolation, cells were stained with fluorescently labeled monoclonal Abs (mAbs) directed against human CD3 (#130-113-138), CD33 (#130-113-350), CD45 (#130-092-880) and CD123 (#130-115-265) (all purchased from Miltenyi Biotec, Bergisch Gladbach, Germany).

### 2.3. Design and Generation of RevCAR T-Cells

Essentially, the RevCAR constructs consist of a signal peptide (SP) derived from human IL-2; the peptide epitope E5B9 or E7B6, derived from the human La/SS-B protein [23] the extracellular hinge (HiD), transmembrane (TMD) and intracellular costimulatory domain (CSD) of the human CD28 (28) connected to the human CD3z (3z) intracellular activating signaling domain (ASD); the peptide T2A (Thosea asigna virus) and the marker EGFP [15]. Furthermore, the Dual-RevCAR vector encodes for two separate RevCARs bicistronically expressed under the control of one promoter. On the one hand, the signaling (SIG) RevCAR-E7B6-3z is flanked by the IL-2-derived SP and the P2A (porcine teschovirus-1) peptide. On the other hand, the costimulatory (COS) RevCAR-E5B9-28 is flanked by the Ig-kappa signal peptide, T2A peptide and EGFP as expression marker [15]. Lentiviral transduction, activation and maintenance of primary T-cells has been performed as previously described [15]. Transduction efficiency of RevCAR T-cells was assessed based on the expression of the marker EGFP.

### 2.4. Determination of Receptor and Antigen Density

Receptor density on RevCAR T-cells and antigen expression on cancer cells were assessed using the QIFIKIT (Agilent, Santa Clara, CA, USA), as already described in detail [15]. Briefly, antigen expression on cancer cells was detected using a purified anti-human CD123 mAb (BD Biosciences, Heidelberg, Germany; #554527) and an in-house produced and purified anti-human CD33 mAb [24]. For detection, PE-conjugated anti-mouse IgG mAb was used (BioLegend, San Diego, CA, USA; #405307).

### 2.5. Design, Expression and Purification of RevTMs

Recently, our group described the general structure of a RevTM [15]. Here, the RevTMs were arranged by cloning the anti-CD33 [14] or the anti-CD123 [17] scFv either to the anti-5B9 or to the anti-7B6 scFv [15], thus generating four different RevTMs. These were further cloned separately into a lentiviral vector and 3T3 cell lines were established to produce the respective RevTM. Purification from cell culture supernatants was achieved via Ni-NTA affinity chromatography [15]. To estimate yield and purity, purified RevTMs were separated via SDS-PAGE and analyzed using Coomassie staining or immunoblotting as described elsewhere [15,18].

### 2.6. Cytokine-Release Assay

Tumor cells were cultured with or without RevCAR T-cells in the presence or absence of RevTMs in a 96-well plate at an effector-to-target cell (e:t) ratio of 5:1 for 18 h or 24 h. Cytokine concentration of TNF-α, IFN-γ, IFN-α, GM-CSF, IL-2, IL-4, IL-5, IL-6, IL-9, IL-10, IL-12, and IL-17A present in cell free supernatants was quantified using the human MACSPlex Cytokine 12 Kit (Miltenyi Biotec) according to manufacturer’s instructions.

### 2.7. Flow Cytometry Analysis

To assess binding of the RevTMs to the RevCAR T-cells and cancer cells, flow cytometry analysis was performed as previously published [15]. For detection, PE- or FITC-conjugated anti-His mAbs (Miltenyi Biotec; #130-120-718 and #120-003-744) were used. Measurements were conducted on a MACSQuant Analyzer^®^ (Miltenyi Biotec) and the acquired data were analyzed using the MACSQuantify Software^®^ (Miltenyi Biotec).

### 2.8. Cytotoxicity Assays

To evaluate the RevCAR T-cell mediated tumor killing, standard chromium (^51^Cr) release, luminescence-based and flow cytometry assays were performed as previously described [13,18,24]. Briefly, triplets of target cells were co-cultured with RevCAR T-cells under several conditions: in the (I) absence, (II) presence or (III) combination of RevTMs at different e:t ratios. For ^51^Cr release assays, MOLM-13 and OCI-AML3 cells were used, for luminescence-based assays MOLM-13-Luc cells were chosen and for flow cytometry assays patient-derived AML blasts were used labeled with eBioscience™ cell proliferation dye eFluor^™^670 (Thermofisher Scientific, Germany). AML cell lysis was determined at increasing RevTM concentrations in the co-culture cytotoxicity assays to calculate the half-maximal effective concentration (EC_50_) using GraphPad Prism 8.

### 2.9. Tumor Xenograft Model and Optical Imaging

All animal procedures have been approved by the local ethics committee for animal experiments (Landesdirektion Dresden, 24–9165.40-4/2013, 24.9168.21–4/2004-1) and were performed at the Helmholtz-Zentrum Dresden-Rossendorf (HZDR) in accordance with the German regulations of animal welfare. Five-week-old male NMRI-nude immunodeficient mice (Rj:NMRI-Foxn1nu/nu) (Janvier Labs, Le Genest-Saint-Isle, France) were randomly (unblinded) assigned to experimental groups of five animals and housed in a pathogen free facility with 12 h light/dark cycle. The health status of the mice was supervised daily by husbandry personnel. To assess in vivo functionality of RevCAR T-cells, 1 × 10^6^ MOLM-13-Luc cells were injected subcutaneously alone or mixed with RevCAR T-cells (1 × 10^6^) and RevTM (17 µg) in a total volume of 100 µL PBS (Thermofisher Scientific). The optical imaging was performed with the In-Vivo Xtreme (Bruker, Nehren, Germany) as described before [15,25].

### 2.10. Statistics

GraphPad Prism 8 (GraphPad Software Inc.) was used to statistically analyze the data. As indicated in the figure legends either one-way or two-way ANOVA with Tukey’s, Dunnett’s or Bonferroni’s multiple comparisons test was used for statistical analysis. *p* values below 0.0332 were considered significant: *p* < 0.0332 (*), *p* < 0.0021 (**), *p* < 0.0002 (***). Data are shown as mean values ± SEM or SD.

## 3. Results

### 3.1. Design and Generation of the RevCAR Platform Targeting CD33 or CD123

Recently, we have introduced the novel versatile adaptor RevCAR platform for T-cell-based cancer immunotherapy. Firstly, proof-of-concept was successfully demonstrated targeting antigens expressed on solid tumors [15]. The purpose of this study was to show the flexibility of the RevCAR platform to be adapted to target myeloid malignancies like AML. As previously published [15] and shown in Figure 1, the RevCAR platform consists of T-cells genetically modified to express RevCARs comprising either the extracellular peptide epitope E5B9 or E7B6. Comparable to second generation CARs, RevCARs contain the CD28 TMD, the intracellular CD28 CSD and the CD3z ASD. Since RevCARs lack an antigen-binding moiety, they require the adaptor molecule named RevTM to recognize malignant cells (Figure 1a). For redirection of RevCAR-E5B9-28/3z or RevCAR-E7B6-28/3z T-cells against CD33 or CD123 expressing AML blasts, we have developed four novel RevTMs, named RevTM CD33-5B9, CD123-5B9, CD33-7B6 or CD123-7B6 (Figure 1a,b). These RevTMs are bispecific Abs (bsAbs) that consist of two scFvs, the first one with specificity for the RevCAR epitope E5B9 or E7B6 and the second one directed against the AML associated antigen CD33 or CD123. After cloning of the DNA sequences encoding the respective RevTM into lentiviral vectors, stable eukaryotic cell lines were established by lentiviral transduction to permanently express and secrete the RevTMs into the cell culture supernatant. Using Ni-NTA affinity chromatography, full length recombinant RevTMs were purified via their C-terminal His tag and analyzed after SDS-PAGE on Coomassie stained gels and on immunoblots detected with an anti-His mAb (Figure 1c,d).

### 3.2. Binding Capability of Bispecific RevTMs to RevCARs and AML Target Antigens

**Figure 2:**
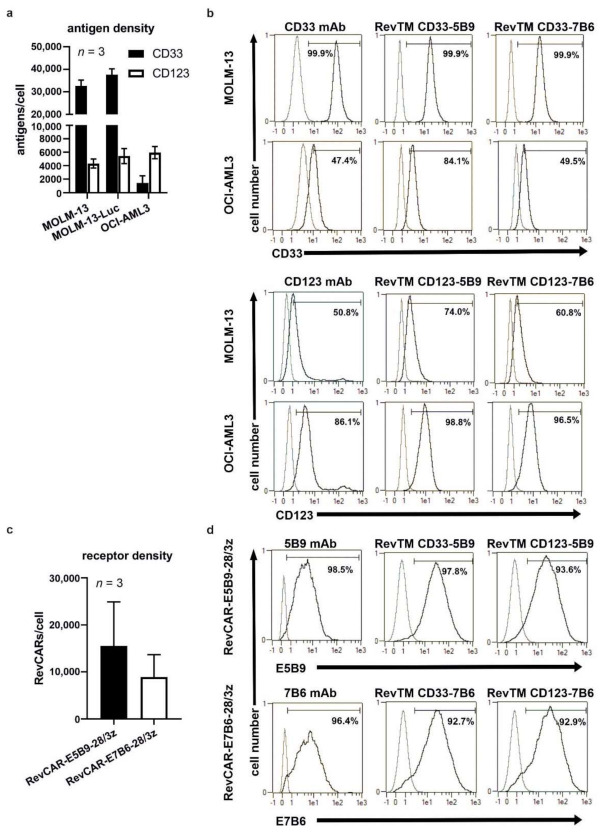
**Expression of target antigens and RevCARs and binding capability of RevTMs.** (**a**) Number of tumor-associated antigens CD33 or CD123 presented on the surface of MOLM-13, Luciferase (Luc) expressing MOLM-13-Luc or OCI-AML3 cells is shown for three individual experiments as mean ± SD. (**b**) Expression of CD33 and CD123 on MOLM-13 and OCI-AML3 cells was confirmed by flow cytometry after staining with APC-conjugated anti-CD33 mAb or PE-conjugated anti-CD123 mAb. Binding of His tagged RevTMs on AML cells was detected via FITC-conjugated anti-His mAb. (**c**) Number of RevCAR-E5B9-28/3z or RevCAR-E7B6-28/3z on RevCAR modified T-cells is quantified for three independent T-cell donors and represented as mean ± SD. (**d**) Expression of RevCAR-E5B9-28/3z or RevCAR-E5B9-28/3z on modified T-cells was confirmed by staining with anti-E5B9 mAb or anti-E7B6 mAb and PE-conjugated anti-mouse-IgG mAb. Binding of indicated RevTMs to RevCAR-E5B9-28/3z or RevCAR-E5B9-28/3z presenting T-cells was detected using PE-conjugated anti-His mAb. (**b**,**d**) Stained cells (black lines) and respective controls (grey lines) are presented as histograms. Percentage of positively stained cells is indicated.

The first step to prove functionality of the novel RevTMs was to analyze binding capability of the bispecific scFv-based RevTMs on the one hand towards CD33 or CD123 expressed on the AML cell lines MOLM-13 and OCI-AML3, and on the other hand towards the RevCAR-E5B9-28/3z or RevCAR-E7B6-28/3z expressed on genetically modified T-cells. This was accomplished by performing cell surface staining using flow cytometry. As a prerequisite, expression of the antigens CD33 or CD123 on the AML cell lines MOLM-13 and OCI-AML3 was confirmed and quantitatively determined using an anti-CD33 mAb and anti-CD123 mAb, respectively (Figure 2a,b). Moreover, analysis of the antigen expression pattern enables a detailed characterization of the different AML cell lines that were used as target cells for subsequent functional assays. In general, the MOLM-13 and OCI-AML3 cell lines expressed both markers, CD33 and CD123, but with different abundance and density. With regard to the antigen density level, MOLM-13 cells expressed clearly more CD33 than CD123 on their cell surface. Conversely, OCI-AML3 cells showed a lower surface expression of CD33 compared to CD123, while the level of CD123 was nearly comparable on both cell lines. It is worth mentioning that the recombinant expression of Luc did not affect the expression level of the target antigens on MOLM-13-Luc cells, which were also used as target cells in subsequent functional assays. Furthermore, the expression of RevCAR-E5B9-28/3z or RevCAR-E7B6-28/3z on genetically modified T-cells was proven and the number of receptors was quantified using anti-5B9 and anti-7B6 mAbs, respectively (Figure 2c,d). Most importantly, all generated RevTMs were able to bind to CD33 or CD123 antigens on the AML cell lines MOLM-13 and OCI-AML3 as well as to the RevCARs on the RevCAR-E5B9-28/3z or RevCAR-E7B6-28/3z T-cells as shown in Figure 2b,d.

### 3.3. Cytokine Release from Redirected RevCAR T-Cells

**Figure 3:**
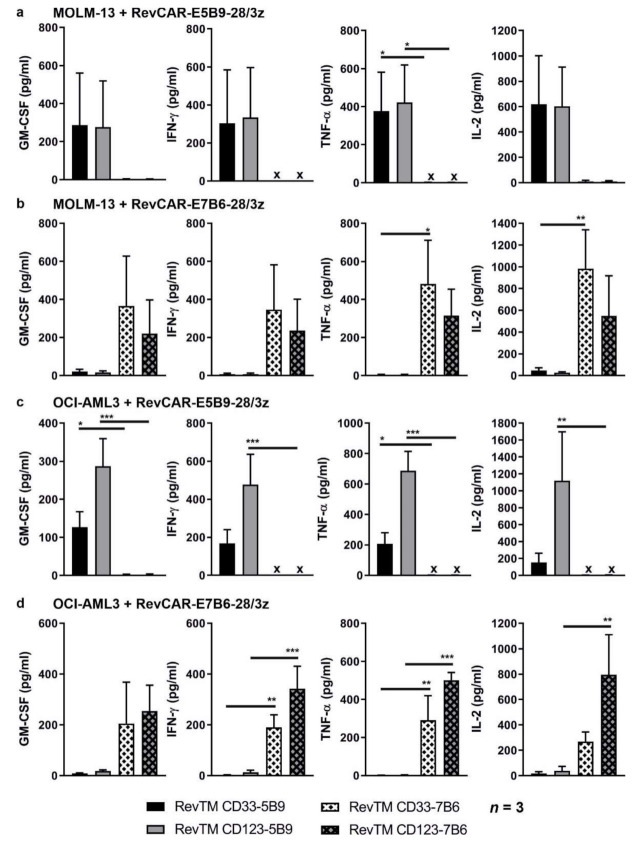
**Cytokine release from redirected RevCAR T-cells.** Release of indicated cytokines from redirected (**a**,**c**) RevCAR-E5B9-28/3z or (**b**,**d**) RevCAR-E7B6-28/3z T-cells co-cultured with (**a**,**b**) MOLM-13 or (**c**,**d**) OCI-AML3 cells at a ratio of 5:1 in the presence of RevCAR epitope matching RevTMs or irrelevant non-matching RevTMs used as negative controls was investigated. Cell culture supernatants were harvested and analyzed. Data acquired for three individual T-cell donors represented as mean ± SD (One-way ANOVA with Tukey’s multiple comparisons test. Significance versus irrelevant RevTMs.). x, not detectable. *p* values below 0.0332 were considered significant: *p* < 0.0332 (*), *p* < 0.0021 (**), *p* < 0.0002 (***).

Upon binding of RevTMs crosslinking AML cells and RevCAR T-cells, RevCAR T-cells are activated resulting in cytokine release and cytotoxic reactivity. In order to prove that, RevCAR-E5B9-28/3z or RevCAR-E7B6-28/3z T-cells were co-cultured with MOLM-13 or OCI-AML3 cells in the presence of RevTMs directed either against the E5B9- or E7B6-RevCARs, respectively. As negative controls, AML cells and RevCAR-E5B9-28/3z T-cells were incubated together with the irrelevant RevTMs CD33-7B6 or CD123-7B6 that are not able to bind to RevCAR-E5B9-28/3z T-cells. Likewise, RevCAR-E7B6-28/3z T-cells were co-cultured with AML cell lines in the presence of the irrelevant RevTMs CD33-5B9 or CD123-5B9. As shown in Figure 3, RevCAR T-cells significantly secreted cytokines after cross-linkage with target cells by the respective matching RevTMs, although high donor variability was observed. These included the secretion of GM-CSF, IFN-γ, TNF-α, and IL-2 among 12 human cytokines tested (Figure 3). Significant release was not detected for the other tested cytokines including, e.g., IL-6. In the presence of irrelevant RevTMs no cytokines were detected in the supernatants.

### 3.4. In Vitro and In Vivo Killing of AML Cell Lines by Redirected RevCAR T-Cells

**Figure 4:**
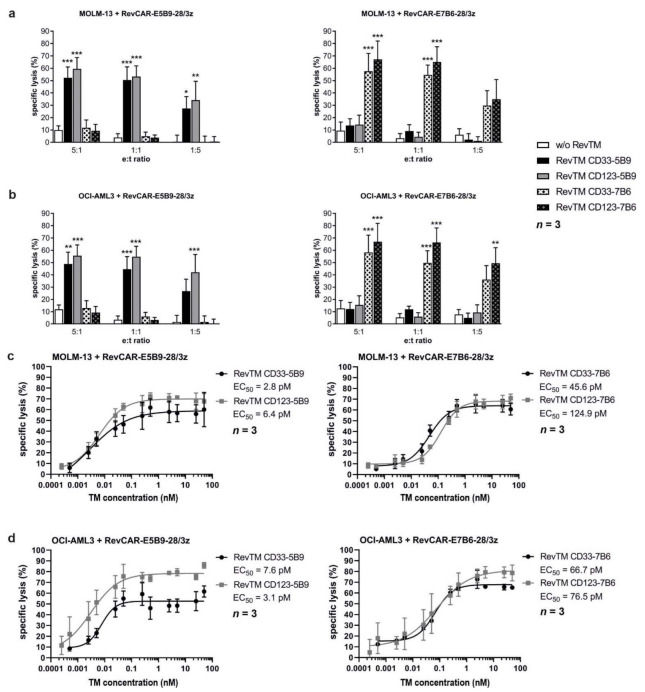
**Lysis of AML cell lines by redirected RevCAR T-cells**. ^51^Cr-labeled (**a**,**c**) MOLM-13 or (**b**,**d**) OCI-AML3 cells were incubated with RevCAR-E5B9-28/3z or RevCAR-E7B6-28/3z T-cells at different effector to target cell (e:t) ratios in the presence or absence of indicated RevTMs. (**a**,**b**) Data are shown for three individual T-cell donors represented as mean ± SEM (Two-way ANOVA with Bonferroni’s multiple comparisons test. Significance was determined as comparison to the setting w/o RevTM.). (**c**,**d**) Effector and target cells were co-cultured at a ratio of 5:1 in the presence of the indicated RevTMs with increasing concentrations to calculate the half-maximal effective concentration (EC_50_) values. Data are shown for three individual T-cell donors represented as mean ± SEM. *p* values below 0.0332 were considered significant: *p* < 0.0332 (*), *p* < 0.0021 (**), *p* < 0.0002 (***).

**Figure 5:**
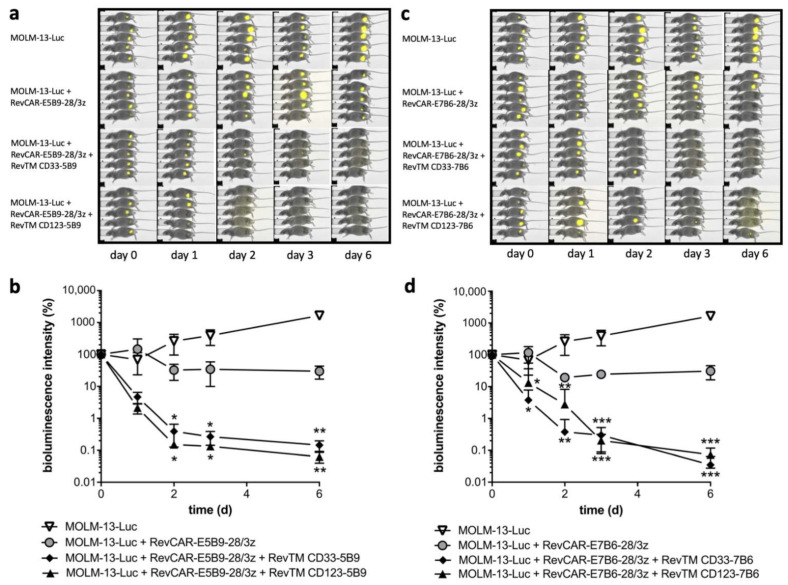
**In vivo killing of MOLM-13 cells by the RevCAR system.** Immunodeficient NMRI-*Foxn1*^nu/nu^ mice were injected with Luciferase (Luc) expressing MOLM-13-Luc cells alone, with MOLM-13-Luc cells together with either (**a**,**b**) RevCAR-E5B9-28/3z or (**c**,**d**) RevCAR-E7B6-28/3z T-cells without RevTMs or with MOLM-13-Luc cells and RevCAR T-cells in the presence of indicated RevTMs. (**a**,**c**) Bioluminescence imaging of anesthetized mice was performed at several time points. (**b**,**d**) The calculated quantitative values of all animals and time points are presented as mean values ± SEM of five animals (one-way ANOVA with Tukey’s multiple comparisons test. Significance versus MOLM-13-Luc + RevCAR-E5B9-28/3z or RevCAR-E7B6-28/3z.). The curves show the relative changes in bioluminescence intensity over the time normalized for each mouse to the starting luminescence at day 0. *p* values below 0.0332 were considered significant: *p* < 0.0332 (*), *p* < 0.0021 (**), *p* < 0.0002 (***).

Most importantly, we were interested in whether the cross-linkage of RevCAR T-cells and target cells mediated by the RevTMs results in AML cell lysis. Therefore, RevCAR T-cells were co-cultured in vitro with MOLM-13 or OCI-AML3 cells at different ratios in the presence or absence of different RevTM concentrations in a ^51^Cr release cytotoxicity assay. Additionally, in a control setting, irrelevant RevTMs that are not able to recognize the RevCAR epitope were used. As shown in Figure 4, RevCAR-E5B9-28/3z or RevCAR-E7B6-28/3z T-cells were effectively redirected by the appropriate cross-linking RevTMs and significantly killed MOLM-13 and OCI-AML3 cells, even at low e:t ratios. In contrast, in the presence of irrelevant RevTMs no specific lysis was observed (Figure 4a,b). Killing of the targeted AML cell lines occurred efficiently even at low RevTM concentrations in the picomolar range and could be controlled by dosing of the RevTM (Figure 4c,d). Besides in vitro experiments, efficient killing of Luc expressing MOLM-13 cells was also observed in experimental mice caused by redirected RevCAR-E5B9-28/3z (Figure 5a,b) or RevCAR-E7B6-28/3z (Figure 5c,d) T-cells in the presence of the matching RevTM targeting either CD33 or CD123. As clearly visible in the bioluminescence images (Figure 5a,c) and confirmed by quantitative analysis (Figure 5b,d), the in vivo anti-tumor effect was significantly higher in comparison to the control mice that were injected with tumor cells together with RevCAR T-cells but without the matching RevTMs. Additionally, there was an effect of the RevCAR T-cells alone against the tumor cells, which can be most probably explained by allogeneic reactions of human T-cells.

### 3.5. Killing of Patient-Derived AML Blasts by Redirected RevCAR T-Cells

**Figure 6:**
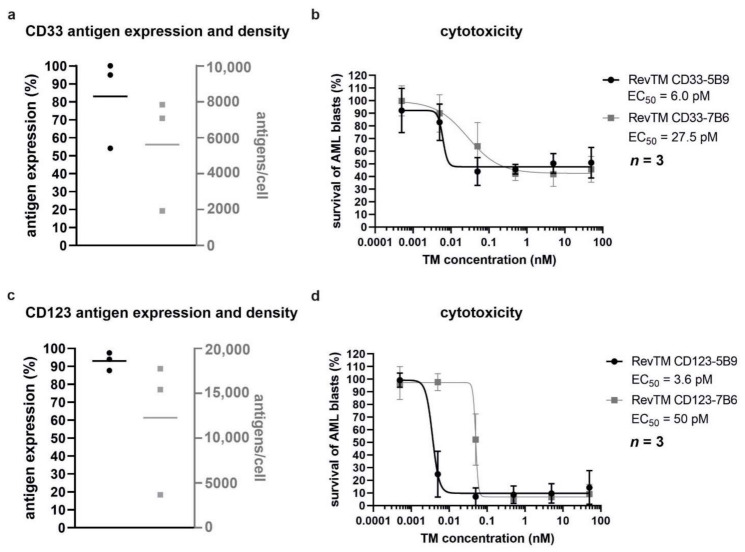
**Killing of patient-derived AML blasts by redirected RevCAR T-cells.** Expression of (**a**) CD33 and (**c**) CD123 on AML blasts from three patients was assessed by flow cytometry after staining with anti-CD33 and anti-CD123 commercial mAbs. Number of tumor-associated antigens (**a**) CD33 or (**c**) CD123 on the surface of patient-derived AML blasts was determined using QiFi kit. (**b**,**d**) RevCAR-E5B9-28/3z or RevCAR-E7B6-28/3z T-cells were co-cultured with the eFluor^™^670-stained patient-derived AML blasts at a ratio of 1:1 in the presence of indicated RevTMs with increasing concentrations in a flow cytometry-based assay, in which the number of living AML blasts was determined. Based on the resulting dose–response curves, half-maximal effective concentration (EC_50_) values were calculated. Data represents three individual T-cell donors and three individual patient-derived AML blasts as mean ± SD.

In order to investigate whether the RevCAR platform can be used to eradicate patient-derived AML blasts, a flow cytometry-based assay was performed. Similarly to the AML cell lines MOLM-13 and OCI-AML3, patient-derived AML blasts expressed both CD33 and CD123 at different density levels (Figure 6a,c). To assess killing, these cells were stained with eFluor^™^670 and co-cultured with RevCAR-E5B9-28/3z or RevCAR-E7B6-28/3z T-cells derived from allogeneic healthy donors in the presence or absence of the appropriate RevTMs at different concentrations. Remarkably, patient-derived AML blasts were significantly killed by redirected RevCAR-E5B9-28/3z or RevCAR-E7B6-28/3z T-cells in the presence of cross-linking RevTMs (Figure 6b,d). The EC_50_ values were in the picomolar range demonstrating the high cytotoxic efficiency of the RevCAR platform. Despite the allogeneic setting used in these experiments, alloreactions of RevCAR T-cells alone against the AML blasts in the absence of any RevTMs were not detected (Supplementary Figure S1). It is worth mentioning that the antigen density varies on AML blasts ranging from around 2000 to 8000 molecules per cell and from around 4000 to 18,000 per cell for CD33 and CD123, respectively (Figure 6a,c). Although the antigen density differs, the maximal lysis and killing efficiency of AML blasts by the RevCAR system was similar (Figure 6b,d). As already observed with AML cell lines (Figure 4), patient-derived AML blasts could be targeted via either CD33 or CD123 confirming the flexibility of the RevCAR system and its suitability for combinatorial targeting.

### 3.6. Combinatorial and Gate Tumor Targeting Using the RevCAR System

**Figure 7:**
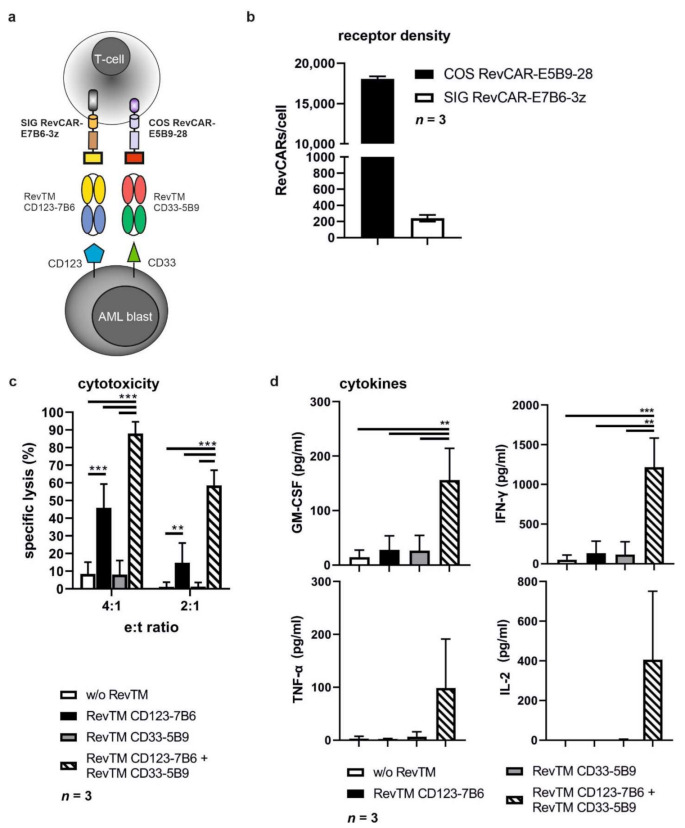
**Combinatorial AND gate targeting of MOLM-13 cells using the Dual-RevCAR system.** (**a**) Schematic overview of the Dual-RevCAR system. Dual-RevCAR T-cells express both the activating signaling SIG RevCAR-E7B6-3z and the costimulatory COS RevCAR-E5B9-28. For complete redirection and activation of Dual-RevCAR-E7B6-3z-E5B9-28 T-cells against AML cells, both RevCARs have to be simultaneously engaged via RevTM CD123-7B6 and RevTM CD33-5B9 recognizing CD123 or CD33, respectively. (**b**) Amount of COS RevCAR-E5B9-28 and SIG RevCAR-E7B6-3z receptors on Dual-RevCAR-E7B6-3z-E5B9-28 T-cells. Three individual T-cell donors are represented as mean ± SD. (**c**,**d**) Proof-of-concept of AND logic gate using the RevCAR system. Dual-RevCAR-E7B6-3z-E5B9-28 T-cells were co-cultured with MOLM-13-Luc cells at indicated e:t ratios in the presence of either the signaling RevTM CD123-7B6, the costimulatory RevTM CD33-5B9 or the combination of both RevTMs. (**c**) In a Luc-based cytotoxicity assay, specific lysis of MOLM-13-Luc cells was calculated for three individual T-cell donors as mean ± SD (Two-way ANOVA with Tukey’s multiple comparisons test. Significance versus w/o RevTM, RevTM CD123-7B6 or RevTM CD33-5B9 alone.). (**d**) Release of the indicated cytokines from redirected Dual-RevCAR-E7B6-3z-E5B9-28 T-cells into the cell culture supernatants of co-cultures was analyzed for three individual T-cell donors as mean ± SD (One-way ANOVA with Tukey’s multiple comparisons test. Significance versus w/o RevTM, RevTM CD123-7B6 or RevTM CD33-5B9 alone). *p* values below 0.0332 were considered significant: *p* < 0.0332 (*), *p* < 0.0021 (**), *p* < 0.0002 (***).

**Figure 8:**
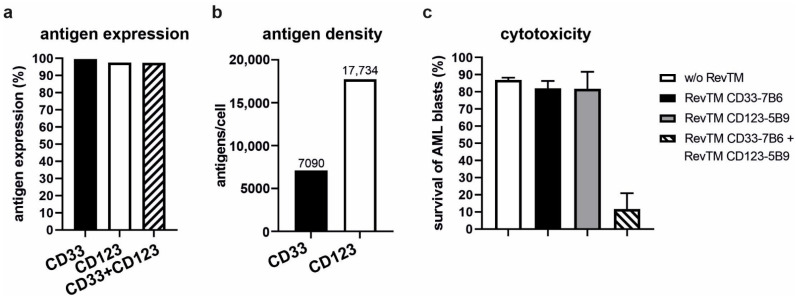
**Combinatorial AND gate targeting of patient-derived AML blasts using the Dual-RevCAR system.** (**a**) Expression of CD33, CD123 and CD33^+^/CD123^+^ on patient-derived AML blasts was assessed by flow cytometry after staining with anti-CD33 and anti-CD123 commercial mAbs. (**b**) Number of tumor-associated antigens CD33 or CD123 on the surface of patient-derived AML blasts was determined using QiFi kit. (**c**) AND gate targeting was assessed on patient-derived AML blasts. Dual-RevCAR-E7B6-3z-E5B9-28 T-cells were co-cultured with the eFluor^™^670-stained patient-derived AML blasts at a ratio of 1:1 in the presence of indicated RevTMs in a flow cytometry-based assay. The SIG RevCAR-E7B6-3z and the COS RevCAR-E5B9-28 receptors on Dual-RevCAR-E7B6-3z-E5B9-28 T-cells were triggered via the RevTM CD33-7B6 and RevTM CD123-5B9, respectively. Data for one sample of patient-derived AML blasts as mean ± SD is shown.

According to our results, we have successfully proven that AML cell lines (Figure 4) and patient-derived AML blasts (Figure 6) expressing either CD33, CD123 or both can be targeted via CD33 or CD123 simply by replacing the respective RevTM. This high flexibility increases the overall anti-tumor effect and enables the targeting of tumor escape variants downregulating or lacking the targeted antigen. In order to reduce on-target/off-tumor reactions and to increase the specificity of the RevCAR T-cell response towards AML blasts, we aimed to establish RevCAR T-cells for a programmable combinatorial targeting of AML blasts co-expressing CD33 and CD123. Therefore, T-cells were genetically modified with a lentiviral vector encoding the SIG and COS RevCARs bicistronically, resulting in Dual-RevCAR T-cells that consequently co-express both receptors (Figure 7a) [15]. In agreement with our previously published data, these Dual-RevCAR T-cells considerably expressed more COS RevCARs than SIG RevCARs on the cell surface of an engineered T-cell (Figure 7b). Dual-RevCAR T-cells were redirected against MOLM-13 cells by RevTM CD123-7B6 triggering the SIG RevCAR-E7B6-3z upon binding to the lower expressed antigen CD123 and by the RevTM CD33-5B9 triggering the COS RevCAR-E5B9-28 upon binding to the higher expressed antigen CD33 (Figure 7c,d). As shown in Figure 7c, under these conditions Dual-RevCAR T-cells achieved a maximum killing of MOLM-13 cells when CD33 and CD123 were recognized simultaneously by both the signaling and costimulatory RevTMs concurrently triggering both signals in the Dual-RevCAR T-cells. Although the CD3z signal alone slightly activated the cytotoxic potential of Dual-RevCAR T-cells, this was considerably lower in comparison to the cell lysis observed in the presence of both the activating and costimulatory signals. As shown in Supplementary Figure S2, the cytotoxic effect of Dual-RevCAR T-cells was increased when the SIG RevCAR-E7B6-3z was triggered by the RevTM CD33-7B6, which targets the higher expressed antigen CD33. Besides cytotoxicity, analysis of cytokine production confirmed that true and complete Dual-RevCAR T-cell activation required both the CD3z and CD28 signal. Cytokines were exclusively and significantly secreted when both signals were triggered in Dual-RevCAR T-cells, in contrast to the control settings without RevTM, or when the activating or the costimulatory RevTMs alone were added (Figure 7d). Moreover, we prove that dual gated targeting using Dual-RevCAR T-cells is not only true for cancer cell lines but also conceivable using patient-derived AML blasts (Figure 8). Stimulation of the SIG RevCAR-E7B6-3z or COS RevCAR-E5B9-28 receptors by the respective RevTMs alone did not promote a cytotoxic response towards the patient-derived AML blasts (Figure 8c). Only simultaneous activation of both signals on Dual-RevCAR T-cells resulted in efficient eradication of these AML blasts. These results provide evidence that AND logic gate using Dual-RevCAR T-cells can be accomplished targeting both AML cancer cell lines as well as patient-derived AML blasts, demonstrating its potential for clinical translation.

## 4. Discussion

The broad clinical application of CAR T-cell technologies for treatment of AML patients is still hindered by challenges particularly related to tumor escape variants, antigen heterogeneity and suitable steering of CAR T-cell activity for safety management. Aiming to tackle such hurdles, adaptor CAR platforms using gated targeting strategies have been developed [26]. One such example is the switchable and controllable RevCAR platform, which has been proven to be suitable for combinatorial targeting of PSCA- and PSMA-positive cancers while retaining flexibility and controllability of engineered CAR T-cells [15]. Herein, we provide proof-of-concept that combinatorial gated targeting could be successfully translated to an AML setting targeting the CD33 and CD123 antigens.

In contrast to conventional second-generation CARs, RevCAR T-cells are engineered to extracellularly express small peptide epitopes derived from the human La/SS-B nuclear protein [23] and are recognized by RevTMs, which are essentially bsAbs with a bispecific T-cell engager (BiTE)-like format specifically binding to the epitopes on RevCAR T-cells and to the tumor-associated antigens. On the one hand, these small adaptor molecules can be simply designed to target any antigen and are easily exchangeable for redirection of RevCAR T-cells to the desired tumor antigens. Moreover, given the similarity of the RevTMs to bsAbs and their small size, a favorable tumor accumulation and tissue penetration are expected along with an in vivo short half-life [23,26,27] allowing a rapid steering of the RevCAR T-cell activity. On the other hand, the reduced size of the RevCARs allows the simultaneous expression of different RevCAR molecules on the surface of one T-cell and subsequently facilitates the separation of costimulatory and activation signals to two CAR constructs envisioning the development of highly flexible gated targeting strategies. In this line of thought, two RevTMs were designed targeting the most common AML antigens CD33 and CD123. In the present work, these have been proven to effectively redirect RevCAR T-cells expressing either E5B9 or E7B6 to eradicate CD33 and CD123-expressing AML cell lines both in vitro and in vivo. According to our data, CD33 targeting seems to be less effective than CD123 targeting using the RevCAR approach. Thus far, we cannot accurately explain why differences between the CD33 and CD123 targeting occurred. However, here we emphasize that both RevTMs targeting either CD33 or CD123 mediate a highly efficient tumor cell lysis in the pM range. In agreement with previous studies using bsAbs, conventional CAR T-cells and adaptor CAR T-cells in combination with differently structured TMs [13,14,15,18,19,20], RevCAR T-cells accordingly released pro-inflammatory cytokines upon RevTM mediated cross-linkage with antigen-expressing cancer cells. Variations in the cytokine profile can be most likely explained by T-cell donor heterogeneity. Aiming clinical translation, additional work was performed using patient-derived material. Similarly, RevCAR T-cells successfully targeted and eliminated AML blasts from patient samples expressing CD33 and CD123 in the presence of the corresponding RevTMs. Given the commonly known heterogeneity of AML blasts, the antigen density varies on the AML blasts. Apparently, a therapeutic window exists for CD33 and CD123 targeting using the RevCAR approach, where no correlation was observed between antigen density level and killing efficacy. Herein, we show that RevCAR T-cell activity can be switched-on/off and steered by dosing of RevTMs. Switchability and controllability are improvements of adaptor universal CAR platform technologies to avoid acute and long-term side effects of conventional CAR T-cell therapy. Since CD33 and CD123 are not exclusively expressed on AML blasts but also on progenitor and mature hematopoietic cells of the myeloid lineage [11], conventional CD33- and CD123-specific CAR T-cells mediate impressive anti-tumor responses but also bear the risk for long-lasting cytopenias [12,28]. As shown in the breakthrough study using the CD123-specific UniCAR approach (which is similar to RevCARs) in relapsed/refractory AML demonstrating promising anti-tumor responses, myelosuppression can be immediately recovered after withdrawal of adaptor TMs providing proof-of-concept for a rapid off-switch of effector adaptor CAR T-cells for the first time in humans enabling an advanced safety management [12]. Given the flexibility of the RevCAR system to target different tumor entities by easily replacing RevTMs with different specificities, a combinatorial gated approach could be established and successfully used to target CD33 or CD123 on the surface of patient-derived AML blasts. This approach is of particular importance to overcome existing or treatment related tumor escape variants and to deal with cancer heterogeneity.

In this line of thought, another step forward within the translational setting would be to design and benefit from an AND logic gate in order to increase tumor specificity and reduce on-target/off-tumor toxicities. This type of logic gate, however, requires a fine-tuning of different features involved in such kind of strategies. These include, e.g., the affinity of the adaptor molecules to both the tumor antigens and adaptor CAR T-cells, adaptor CAR and tumor antigen density, and an optimal balance between the strength of the costimulatory and activation signals [29,30,31,32,33]. As already mentioned in our previous publication, a huge series of RevCARs containing different HiDs, TMDs and SDs were constructed and tested with respect to expression, dimerization, functionality, and suitability for AND gate targeting [15]. Despite the challenges, our group recently demonstrated that AND logic gate could be successfully applied using Dual-RevCAR T-cells to target two antigens associated with prostate cancer [15]. Likewise, in the present work, we have demonstrated that the Dual-RevCAR system can be applied to tackle common myeloid malignancies, such as AML, based on an AND gate targeting of the most common antigens CD33 and CD123. For that and as previously mentioned, key parameters needed to be fulfilled. These particularly included the balance between receptor density and strength of the costimulatory and activation signals on the Dual-RevCAR T-cells, and use of the most suitable combination of the respective RevTMs. This optimal setting was indeed accomplished with the considerably higher expression of the COS RevCAR-E5B9-28 receptors on the surface of Dual-RevCAR T-cells in comparison to the SIG RevCAR-E7B6-3z and the combination of the herein used RevTMs. Furthermore, the density level of the targeted antigens is critical for true AND gate targeting. As shown for AML cell lines, Dual-RevCAR T-cells follow the AND gate logic when the SIG RevCAR-E7B6-3z is triggered by the RevTM targeting the lower expressed antigen and the COS RevCAR-E5B9-28 is triggered by the RevTM targeting the higher expressed antigen. Vice versa, when the SIG RevCAR-E7B6-3z is engaged via the RevTM targeting the higher expressed antigen, the effect of the signaling RevCAR alone is increased in a way that true AND gate targeting cannot be achieved anymore. These conditions led to the establishment of a holistic AND gated targeting, which culminated in the activation of the Dual-RevCAR T-cells specifically promoting the release of pro-inflammatory cytokines and killing of AML cell lines expressing both antigens of interest. Some cytotoxicity of Dual-RevCAR T-cells was triggered by the SIG RevCAR-E7B6-3z alone cross-linked with target antigen via the respective RevTM. This effect was however not unexpected, since the intracellular portion of the signaling RevCARs are structural and functional comparable to conventional CARs of the first generation which are known to be able to mediate effective tumor cell killing. Nevertheless, this activation signal alone was not sufficient to induce the production of pro-inflammatory cytokines and significantly higher killing was obtained using the appropriate combination of both RevTMs to trigger both signals showing true AND gated targeting. Importantly, such logic gate was furthermore achieved using patient-derived material demonstrating its forthcoming therapeutic relevance.

In recent years, work from several groups has been done aiming the design of modular gated strategies using CAR T-cells targeting different tumor entities. These modular systems reported up to now include the use of, e.g., zipCARs, biotin avidin systems, yeast-derived intermediary molecules among others [26,30,31,34,35]. However, problems associated with, e.g., immunogenicity, lack of controllability or versatility still hinder certain modular approaches as only some of them have been proven to be suitable for logic gated targeting and eventually move forward to clinical trials [26,30,31]. Given the proven unique features and versatility of the RevCAR system and aiming treatment of AML patients, to the best of our knowledge this is the first pre-clinical report and proof-of-concept of successful targeting of AML cells using logic gated strategies.

## 5. Conclusions

In conclusion, we hereby demonstrated the preclinical versatility, controllability and efficacy of the RevCAR platform for the targeting of AML. Furthermore, this is the first report and proof-of-concept of successful targeting of AML cells using logic gated strategies, supporting its potential and suitability for future clinical translation.

## Data Availability

The data presented in this study are available on request from the corresponding author.

## Supplementary Materials

The following are available online at https://www.mdpi.com/article/10.3390/cancers13194785/s1, Figure S1: Killing of patient-derived AML blasts by RevTM-redirected RevCAR T-cells. Figure S2: Combinatorial targeting of MOLM-13 cells using the Dual-RevCAR system. Figure S3: Whole western blot.
