# Supplementary material for: Targeting Acute Myeloid Leukemia Using the RevCAR Platform: A Programmable, Switchable and Combinatorial Strategy
Source: Cancers (Basel). 2021 Sep 24;13(19):4785. doi: 10.3390/cancers13194785 (PMC8508561; doi:10.3390/cancers13194785)
Supplement: Supplementary file 1 [file cancers-13-04785-s001.zip › cancers-1368336-SI.pdf]

## Supplementary Information

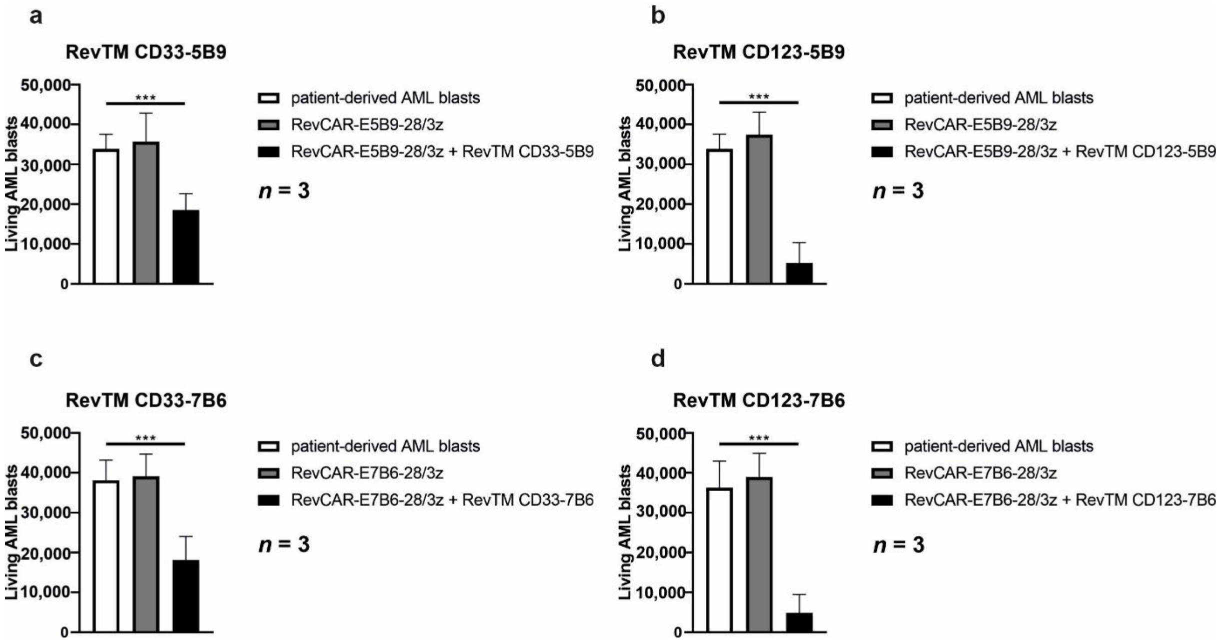


**Supplementary Figure S1. Killing of patient-derived AML blasts by RevTM-redirected RevCAR T-cells.** Patient-derived AML blasts stained with eFluor™670 were cultured alone or together with (a, b) RevCAR-E5B9-28/3z or (c, d) RevCAR-E7B6-28/3z T-cells at a ratio of 1:1 in the absence or presence of indicated RevTMs at a saturation concentration of 50 nM. After incubation of 24 h, the number of living AML blasts was determined via flow cytometry. Data represents three individual T-cell donors and three individual patient-derived AML blasts as mean ± SD (One-way ANOVA with Tukey's multiple comparisons test. Significance versus patient-derived AML blasts.).

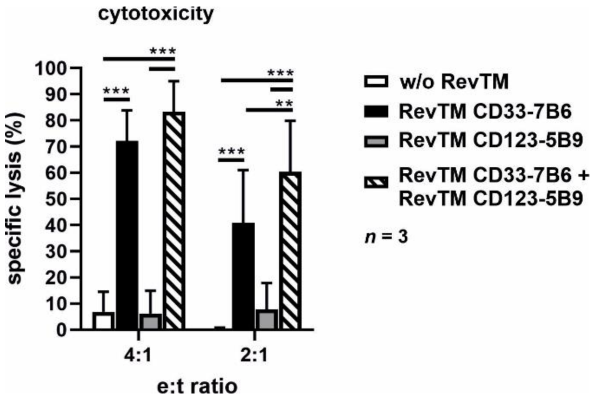


**Supplementary Figure S2. Combinatorial targeting of MOLM-13 cells using the Dual-RevCAR system.** Dual-RevCAR-E7B6-3z-E5B9-28 T-cells were co-cultured with MOLM-13-Luc cells at indicated e:t ratios in the presence of either the signaling RevTM CD33-7B6, the costimulatory RevTM CD123-5B9 or the combination of both RevTMs in a Luc-based cytotoxicity assay. Specific lysis of MOLM-13-Luc cells was calculated for three individual T-cell donors as mean ± SD (Two-way ANOVA with Tukey's multiple comparisons test. Significance versus w/o RevTM, RevTM CD33-7B6 or RevTM CD123-5B9 alone.).

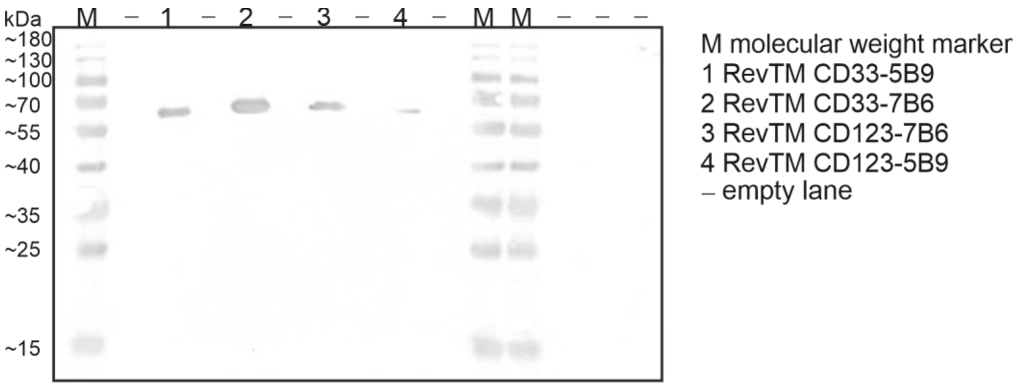


**Supplementary Figure S3. Whole western blot.** After purification via histidine tag (His), RevTMs were separated by SDS-PAGE and analyzed using immunodetection after blotting on nitrocellulose membrane via anti-His Ab and AP-conjugated anti-mouse Ab.
